# Intestinal Microbiota Shifts towards Elevated Commensal *Escherichia coli* Loads Abrogate Colonization Resistance against *Campylobacter jejuni* in Mice

**DOI:** 10.1371/journal.pone.0035988

**Published:** 2012-05-01

**Authors:** Lea-Maxie Haag, André Fischer, Bettina Otto, Rita Plickert, Anja A. Kühl, Ulf B. Göbel, Stefan Bereswill, Markus M. Heimesaat

**Affiliations:** 1 Department of Microbiology and Hygiene, Charité - University Medicine Berlin, Berlin, Germany; 2 Department of Internal Medicine, Rheumatology and Clinical Immunology/Research Center ImmunoSciences (RCIS), Charité - University Medicine Berlin, Berlin, Germany; University of Aberdeen, United Kingdom

## Abstract

**Background:**

The zoonotic pathogen *Campylobacter jejuni* is a leading cause of bacterial foodborne enterocolitis in humans worldwide. The understanding of immunopathology underlying human campylobacteriosis is hampered by the fact that mice display strong colonization resistance against the pathogen due to their host specific gut microbiota composition.

**Methodology/Principal Findings:**

Since the microbiota composition changes significantly during intestinal inflammation we dissected factors contributing to colonization resistance against *C. jejuni* in murine ileitis, colitis and in infant mice. In contrast to healthy animals *C. jejuni* could stably colonize mice suffering from intestinal inflammation. Strikingly, in mice with *Toxoplasma gondii*-induced acute ileitis, *C. jejuni* disseminated to mesenteric lymphnodes, spleen, liver, kidney, and blood. In infant mice *C. jejuni* infection induced enterocolitis. Mice suffering from intestinal inflammation and *C. jejuni* susceptible infant mice displayed characteristical microbiota shifts dominated by increased numbers of commensal *Escherichia coli*. To further dissect the pivotal role of those distinct microbiota shifts in abrogating colonization resistance, we investigated *C. jejuni* infection in healthy adult mice in which the microbiota was artificially modified by feeding live commensal *E. coli*. Strikingly, in animals harboring supra-physiological intestinal *E. coli* loads, colonization resistance was significantly diminished and *C. jejuni* infection induced enterocolitis mimicking key features of human campylobacteriosis.

**Conclusion/Significance:**

Murine colonization resistance against *C. jejuni* is abrogated by changes in the microbiota composition towards elevated *E. coli* loads during intestinal inflammation as well as in infant mice. Intestinal inflammation and microbiota shifts thus represent potential risk factors for *C. jejuni* infection. Corresponding interplays between *C. jejuni* and microbiota might occur in human campylobacteriosis. Murine models introduced here mimick key features of human campylobacteriosis and allow for further analysis of immunological and molecular mechanisms of *C. jejuni* – host interactions.

## Introduction

*Campylobacter (C.) jejuni* continues to be the most frequently reported causative agent of bacterial gastroenteritis in humans worldwide [1], [2]. The gram-negative bacteria form part of the commensal flora in many wild and domestic animals. Zoonotic transmission from livestock animals via consumption of contaminated meat products is the most common source for human *C. jejuni* infections [3], [4], [5]. Infected patients present a broad range of symptoms including abdominal pain, watery or bloody diarrhea, and fever. Histological examination of affected intestinal tissues commonly reveals crypt abscesses, ulcerations, and elevated numbers of immune cells in the colon *in situ* [6], [7], [8], [9]. Although the infection is self-limiting in most cases, long-term sequelae such as reactive arthritis, gastrointestinal and neurological disorders have been reported in susceptible hosts [10], [11]. *C. jejuni* infection correlates with reactivation of inflammatory bowel diseases (IBD), Miller-Fisher-Syndrome and Guillian-Barré-Syndrome [10], [11]. The high prevalence of campylobacteriosis poses a significant health burden and accentuates the need for further unravelling the underlying pathogenesis of *C. jejuni* infections. The lack of understanding mainly stems from the scarcity of suitable experimental *in vivo* models of human infection [12]. Although chickens allow for the investigation of colonization and transmission, intestinal disease manifestations such as enteritis and diarrhea are absent [12], [13]. Murine models of *C. jejuni* infection have the disadvantage of sporadic colonization, absence of clinical signs of disease and/or lack of intestinal immunopathology [6], [12]. This is in part due to the colonization resistance against *C. jejuni* exhibited by adult mice older than two months (commonly used in experimental settings) harboring a conventional intestinal microbiota [12]. Results from the 1980s demonstrated that colonization resistance was abrogated in isolator raised germfree (GF) mice. In these animals *C. jejuni* colonized the entire gastrointestinal (GI) tract and induced clinical signs of enterocolitis including granulocyte infiltrates, bloody diarrhea, and humoral immune responses [14], [15], [16]. More recent investigations showed that colonization resistance is mainly caused by the host-specific intestinal microbiota composition [17], [18]. Previous work in our group revealed that colonization resistance of mice against *C. jejuni* is abrogated in gnotobiotic (GB) mice generated by quintuple antibiotic treatment as well as in GB mice replenished with human flora (hfa) [17]. In contrast to isolator raised germfree mice, however, GB and hfa animals display a fully established immune system. The finding that GB mice replenished with murine flora were completely resistant to *C. jejuni* proved that colonization resistance is caused by the host-specific microflora composition [17]. Most strikingly, *C. jejuni* infected GB and hfa mice displayed characteristic inflammatory responses seen in human campylobacteriosis [17]. The major role of the microflora composition in colonization resistance against *C. jejuni* was further supported by the recent finding that obesity caused by both feeding human “Western style” cafeteria diet (CAF) as well as leptin deficiency in *ob/ob^−/−^* mice rendered mice susceptible to *C. jejuni* infection [18]. Interestingly, quantitative analysis of main gut bacterial groups revealed 2–3 orders of magnitude higher loads of commensal *E. coli* in *C. jejuni* susceptible animals in all these models as a common feature as compared to respective resistant controls [17], [18]. Recent studies on *Salmonella* enterica also point towards a pivotal role of microbiota composition in host susceptibility to enteropathogenic bacteria [19], [20]. Mice displaying high commensal *E. coli* densities were more susceptible to *S. enterica* induced gut inflammation [19], [20], [21]. We have previously shown that murine models of acute small as well as large intestinal inflammation were associated with tremendous changes of the intestinal microbiota with decreasing species diversity and lactobacilli loads and an intestinal overgrowth with commensal *E. coli* and *Bacteroides/Prevotella* spp. [22], [23], [24], [25]. We could further show that TLR-4-signalling of *E. coli* LPS initiated and perpetuated acute ileitis and colitis in mice [23], [24]. Thus, these experimental models were mimicking immunopathological key features of human IBD Crohn’s disease (CD) and ulcerative colitis (UC). The exact etiology of IBD remains unknown and the pathogenesis seems to be multifactorial, involving the complex interaction between the intestinal microflora, host immune and genetic factors as well as environmental stimuli [26], [27], Gram-negative bacteria such as *E. coli* and *Bacteroides* spp. have been reported to accumulate at inflamed tissue sites in IBD patients with active intestinal inflammation, and further potentiate immunopathology by translocation via microlesions and ulcerations [28], [29]. It is under current debate whether IBD patients might be more susceptible to intestinal pathogens such as *Campylobacter* or *Salmonella* spp. and whether acute bacterial enterocolitis is one of the factors inducing or exacerbating IBD in susceptible individuals [26], [27], [30]. Results from population-based studies point towards a potential role of enteric *Campylobacter* or *Salmonella* infections in IBD development [31], [32], [33].

In the present study, we investigated colonization resistance in mice displaying an altered gut microbiota composition under conditions of acute or chronic intestinal inflammation as well as in infant mice after weaning (lacking signs of intestinal inflammation) having all elevated intestinal *E. coli* loads in common. Finally we asked the question whether feeding healthy adult mice with live *E. coli* and thus artificially elevating the enterobacteria population within the commensal gut microbiota towards supra-physiological loads might overcome colonization resistance and renders the animals susceptible to *C. jejuni*. The results clearly demonstrate that colonization resistance is completely abrogated under conditions of elevated *E. coli* loads within the commensal intestinal microbiota. Following infection, *C. jejuni* induced pro-inflammatory immune responses mimicking key features of human campylobacteriosis. Taken together, the murine *C. jejuni* infection models presented here proved to be suitable for reproducible investigations of factors that predispose *C. jejuni* colonization and trigger the onset and progress of infection and immunopathology.

## Results

### *T. gondii*-induced Acute Ileitis Abrogates Colonization Resistance of Mice Against *C. jejuni* Infection

**Figure 1:**
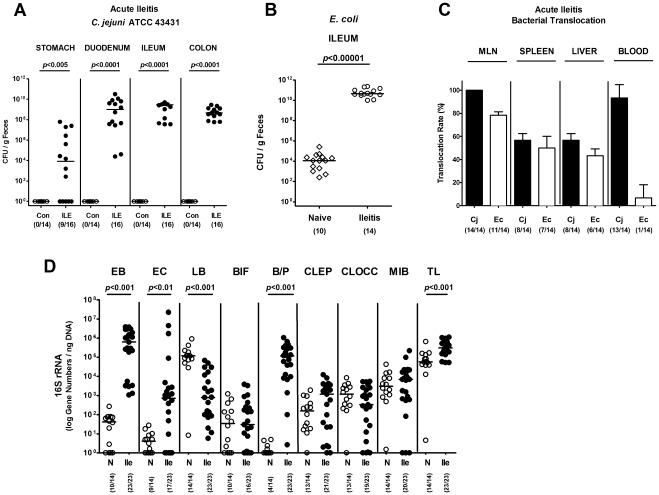
*C. jejuni* colonization along the gastrointestinal tract of mice with *T. gondii*-induced acute ileitis. Acute ileitis was induced in conventionally colonized 3-months-old C57BL/6 mice by oral infection with 100 cysts of *Toxoplasma gondii* ME49 strain. Four days following ileitis induction, mice were orally infected with *C. jejuni* strain ATCC 43431 as described in Methods. (**A**) At day 7 p.i. when *T. gondii* infected mice display severe ileitis (ILE) *C. jejuni* densities in distinct compartments of the gastrointestinal tract were determined by quantification of live *C. jejuni* in luminal samples taken from stomach, duodenum, ileum, and colon by cultural analysis (CFU, colony forming units; solid circles) and compared to control mice without ileitis (Con). (**B**) *E. coli* loads in luminal ileum samples of mice with *T. gondii*-induced acute ileitis (day 7 p.i.; open circles) were compared to naïve controls (open diamonds). (**C**) Relative translocation frequencies (%) of live *C. jejuni* 43431 ATCC (Cj, black bars) and *E. coli* (Ec, open bars) were determined in *ex vivo* biopsies of mesenteric lymphnodes (MLN), spleen, liver, and blood derived from mice with *T. gondii*-induced acute ileitis at day 7 p.i. by culture in enrichment broths. (**D**) A complex molecular analysis of the ileal microbiota composition was assessed in mice with *T. gondii*-induced acute ileitis at day 7 p.i. (ILE; solid circles) and compared to naïve control animals (N; open circles). Main gut bacterial groups were quantified by Real-Time-PCR analyses amplifying bacterial 16S rRNA variable regions and 16S rRNA gene numbers/ng DNA derived from luminal ileal samples: *Enterobacteriaceae* (EB), Enterococci (EC), Lactic acid bacteria (LB), *Bifidobacteria* (BIF), *Bacteroides/Prevotella* spp. (BP), *Clostridium leptum* group (CLEP), *Clostridium coccoides* group (CLOCC), Mouse intestinal bacteroidetes (MIB), and total eubacterial load (TL). Numbers of animals harboring *C. jejuni* or *E. coli* out of the total number of analyzed animals are given in parentheses. Medians (black bars), standard deviations and significance levels (*P*-values) determined by Mann-Whitney-U test are indicated. Data shown were pooled from at least three independent experiments.

Colonization resistance against *C. jejuni* exerted by mice harbouring a conventional intestinal microbiota can be overcome by modification of the gut microbiota composition. We therefore investigated *C. jejuni* infection capacities in mice with acute or chronic intestinal inflammation displaying distinct microbiota shifts towards higher intestinal enterobacteria loads. Three-months-old adult C57BL/6 mice harboring a conventional gut microbiota were infected perorally with *T. gondii* in order to induce acute ileitis. Four days thereafter, the time point when initial histopathological changes in the ileum mucosa can be observed, mice were orally subjected to 10^9 ^CFU *C. jejuni* ATCC 43431 by gavage. At day 7 following *T. gondii* infection, mice suffered from acute ileitis and were prone to death. Interestingly, by that time (i.e. 3 days following *C. jejuni* ATCC 43431 infection) *C. jejuni* ATCC 43431 could be cultured from the entire GI tract of diseased, but not healthy mice with high loads of 10^9^ CFU/g in duodenum, ileum and colon (**Fig. 1A**). Notably, the *E. coli* concentrations in the ileum lumen had increased by more than 6 orders of magnitude up to more than 10^10^ CFU/g during ileitis development (**Fig. 1B**). Live *C. jejuni* ATCC 43431 and commensal *E. coli* translocated into MLNs draining the small intestine (100±0% and 78.6±7.1%, respectively), spleen (57.1±1.49% and 50.0±0.68%, respectively) and liver (57.1±0% and 42.9±0%, respectively) (**Fig. 1C**). Strikingly, in 92.9±0.68%, but only in 7.1±0.7% of mice with acute ileitis following *T. gondii* infection, live *C. jejuni* ATCC 43431 and *E. coli*, respectively, could be cultured from cardiac blood indicating *C. jejuni* bacteremia (**Fig. 1C**). A molecular survey of the ileum microbiota composition revealed that concentrations of total eubacteria, enterobacteria, enterococci and *Bacteroides/Prevotella* spp. were increased in *T. gondii*-induced acute ileitis, whereas the lactobacilli population was lower as compared to healthy mice (**Fig. 1D**). Thus, during acute small intestinal inflammation accompanied by distinct intestinal microbiota shifts towards *E. coli* overgrowth in the GI tract colonization resistance against *C. jejuni* is abrogated with hazardous consequences for the host, as indicated by translocation and systemic spread of the pathogen.

### IL-10 Deficient Mice with Chronic Colitis are Susceptible to *C. jejuni* Colonization

**Figure 2:**
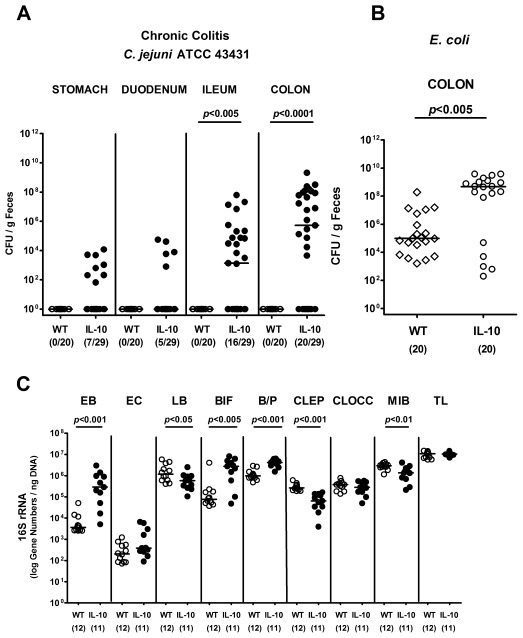
*C. jejuni* colonization along the gastrointestinal tract of IL-10^−/−^ mice with chronic colitis. Conventionally colonized 6-months-old IL-10*^−^* ^/*−*^ mice with chronic colitis were orally infected with *C. jejuni* strain ATCC 43431 as described in Methods. (**A**) At day 14 p.i. *C. jejuni* densities in distinct compartments of the gastrointestinal tract were determined by quantification of live *C. jejuni* in luminal samples (CFU, colony forming units; solid circles) taken from stomach, duodenum, ileum, and colon by cultural analysis and compared to wildtype controls (WT, open circles). (**B**) Furthermore, *E. coli* loads in luminal colonic samples of *C. jejuni* infected IL-10*^−^* ^/*−*^ mice (IL-10, open circles) were compared to healthy wildtype control animals (WT, open diamonds). (**C**) A complex molecular analysis of the colonic microbiota composition was assessed in IL-10*^−^* ^/*−*^ mice with chronic colitis (IL-10; solid circles) and compared to wildtype control animals (WT; open circles). Main gut bacterial groups were quantified by Real-Time-PCR analyses amplifying bacterial 16S rRNA variable regions and 16S rRNA gene numbers/ng DNA derived from luminal colonic samples: *Enterobacteriaceae* (EB), Enterococci (EC), Lactic acid bacteria (LB), *Bifidobacteria* (BIF), *Bacteroides/Prevotella* spp. (BP), *Clostridium leptum* group (CLEP), *Clostridium coccoides* group (CLOCC), Mouse intestinal bacteroidetes (MIB), and total eubacterial load (TL). Numbers of animals harboring *C. jejuni* or *E. coli* out of the total number of analyzed animals are given in parentheses. Medians (black bars) and significance levels (*P*-values) determined by Mann-Whitney-U test are indicated. Data shown were pooled from at least three independent experiments.

Next, we were interested whether colonization resistance against *C. jejuni* is also abrogated in mice with chronic intestinal inflammation accompanied with gut microbiota shifts towards higher commensal enterobacteria loads [34]. To address this question we infected 6-months-old IL-10*^−^* ^/*−*^ mice with chronic colitis harboring a conventional gut microbiota with *C. jejuni* ATCC 43431. Twelve days following oral infection, *C. jejuni* could be detected in the ileum and colon (in 55.2% and 69.0% of infected mice, respectively), but not in the stomach or duodenum of IL-10*^−^* ^/*−*^ mice with colitis, whereas healthy wildtype control animals remained free of *C. jejuni* (**Fig. 2A**). Furthermore, the median *C. jejuni* loads in infected IL-10*^−^* ^/*−*^ mice were more than 4 orders of magnitude lower (10^5^ CFU/g luminal content) when compared to mice with *T. gondii*-induced acute ileitis (**Fig. 1A** **,** **2A**). Of note, uninfected IL-10*^−^* ^/*−*^ mice with colitis harbored 4 orders of magnitude higher *E. coli* counts in their large intestines when compared to healthy wildtype mice (**Fig. 2B**). Interestingly, live *C. jejuni* could be cultured only from less than 5% (3 out of 67) of MLNs derived from infected IL-10*^−^* ^/*−*^ mice, whereas no *C. jejuni* at all translocated into spleen, liver, kidney or cardiac blood at day 12 p.i. (not shown). A molecular survey of the main bacterial groups within the colonic lumen revealed that IL-10*^−^* ^/*−*^ mice with colitis harbored significantly higher numbers of enterobacteria, *Bacteroides/Prevotella* spp. and bifidobacteria, whereas lactobacilli, *Clostridium leptum* group and mouse intestinal bacteroidetes rRNA were lower as compared to wildtype controls, (**Fig. 2C**). Thus, colonization resistance against *C. jejuni* is abrogated by chronic large intestinal inflammation in IL-10*^−^* ^/*−*^ mice accompanied by distinct changes in colonic microbiota composition.

### *C. jejuni* Infection of Infant Mice Harboring a Conventional Intestinal Microbiota

**Figure 3:**
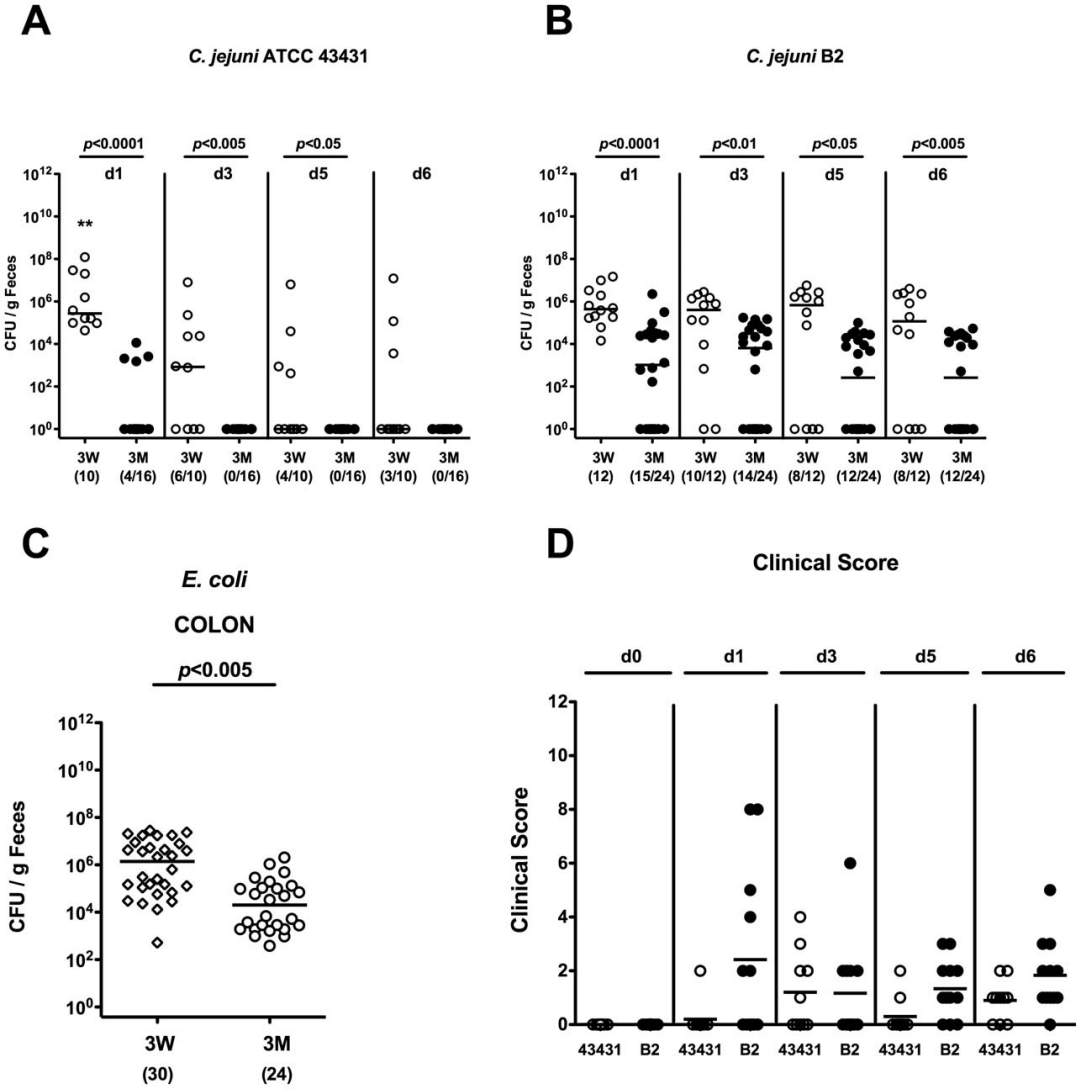
Kinetic analyses of *C. jejuni* colonization in fecal samples of infant mice. Conventionally colonized 3-weeks-old infant (3W; open circles) and 3-months-old adult (3M; solid circles) mice were orally infected with *C. jejuni* strain ATCC 43431 (**A**) or B2 (**B**) as described in Methods. Kinetic analyses of pathogen densities were performed by quantification of live *C. jejuni* in fecal samples by cultural analysis (CFU, colony forming units) at day (d) 6 p.i. ** indicates significant differences (p<0.01) of *C. jejuni* 43431 loads in infected infant mice at d1 versus d3, d5, and d6 p.i. (**C**) *E. coli* loads in colonic samples taken from naïve 3-weeks-old infant mice (3W, open diamonds) were compared to conventionally colonized 3-months-old adult mice (3M, open circles). (**D**) Kinetic analyses of disease activity of conventionally colonized 3-weeks-old infant mice following infection with *C. jejuni* strain ATCC 43431 (43431, open circles, n = 10) or B2 (solid circles, n = 12) were performed applying a standardized clinical score (see Methods). Numbers of animals harboring *C. jejuni* or *E. coli* out of the total number of analyzed animals are given in parentheses. Days past infection, medians (black bars) and significance levels (*P*-values) determined by Mann-Whitney-U test are indicated. Data shown were pooled from at least three independent experiments.

In earlier studies weaned mice harboring a conventional gut microbiota were shown to be susceptible to *C. jejuni* infection [35], [36], [37], [38], [39]. Very recently, we could demonstrate that compared to adult animals, infant mice harbored significantly higher concentrations of *E. coli*, *Bacteroides/Prevotella* spp. and *Clostridium coccoides* whereas lactobacilli, bifidobacteria and *Clostridium leptum* numbers were lower [40]. To further unravel immunopathological consequences of abrogated colonization resistance in infant mice in more detail, we infected 3-weeks-old animals right after weaning with *C. jejuni* strains ATCC 43431 or B2. Adult mice 3 months of age with a common commensal gut flora served as controls. Kinetic analyses of fecal pathogen burden revealed that the infant mice harbored higher *C. jejuni* ATCC 43431 and B2 loads until days 5 and 6 p.i., respectively, when compared to adult animals (**Fig. 3A, B**). Interestingly, *C. jejuni* strain B2, but not strain ATCC 43431, could stably infect infant mice until day 6 p.i., as indicated by 66.7% and 30.0% of culture-positive cases, respectively, with a median load of approximately 10^5^ CFU *C. jejuni* B2/g feces (**Fig. 3A, B**). Feces samples taken from naïve infant mice contained significantly higher numbers of *E. coli* (median 10^6^ CFU/g), as compared to uninfected adult animals (**Fig. 3C**). Thus, these data provide evidence that *E. coli* might be causatively involved in the abrogation of murine colonization resistance against *C. jejuni*.

The analysis of the clinical outcome revealed that acute campylobacteriosis was self-limiting in infant mice. A cumulative score addressing the clinical condition and occurrence of bloody diarrhea in infected animals revealed that either *C. jejuni* strain induced enterocolitis within 24 hours p.i. remaining on a comparable level until day 6 p.i. (**Fig. 3D**). *C. jejuni* infected infant mice were not severely compromised clinically but developed mild diarrhea with haemoccult-positive feces irrespective of the *C. jejuni* strain analyzed. Furthermore, no live *C. jejuni* could be cultured from MLNs, spleen, liver, kidney or cardiac blood at day 6 following infection with either strain (not shown).

### Pro-inflammatory Immune Responses Following *C. jejuni* Infection in Infant Mice

**Figure 4:**
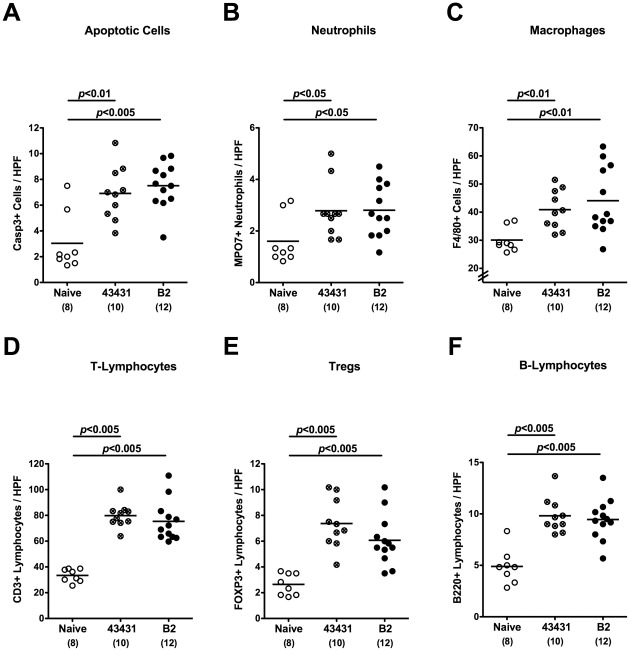
Inflammatory and immune cell responses following *C. jejuni* infection of infant mice. Conventionally colonized 3-weeks-old infant mice were orally infected with *C. jejuni* strain ATCC 43431 (crossed circles) or B2 (solid circles) as described in Methods. The average numbers of apoptotic cells (positive for caspase-3, panel **A**), neutrophilic granulocytes (neutrophils, positive for MPO-7, panel **B**), macrophages (positive for F4/80, panel **C**), T-lymphocytes (positive for CD3, panel **D**), regulatory T-cells (Tregs, positive for FOXP3, panel **E**) and B-lymphocytes (positive for B220, panel **F**) from at least six high power fields (HPF, ×400 magnification) per animal were determined microscopically in immunohistochemically stained colon sections at day 6 p.i. Uninfected mice (Naïve, open circles) served as negative controls. Numbers of analyzed animals are given in parentheses. Means (black bars) and levels of significance (*P*-values) determined by the Student’s *t*-test are indicated. Data shown are representative for three independent experiments.

**Figure 5:**
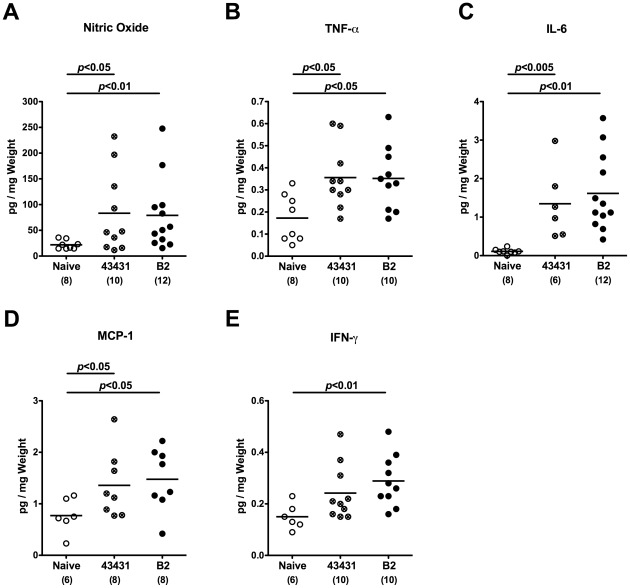
Pro-inflammatory cytokine responses in the colon of *C. jejuni* infected infant mice. Conventionally colonized 3-weeks-old infant mice were orally infected with *C. jejuni* strain ATCC 43431 (43431, crossed circles) or B2 (solid circles) as described in Methods. Concentrations (pg per mg colon) of (**A**) nitric oxide, (**B**) TNF-α, (**C**) IL-6, (**D**) MCP-1, and (**E**) IFN-γ were determined in supernatants of *ex vivo* colon cultures at day 6 p.i. by Griess reaction or CBA-ELISA, respectively. Uninfected mice (Naïve, open circles) served as negative controls. Numbers of analyzed animals are given in parentheses. Means (black bars) and levels of significance (*P*-values) as compared to the respective group (determined by Student’s *t*-test) are indicated. Data shown are representative for three independent experiments.

Since human campylobacteriosis is accompanied by an influx of immune cells to the inflamed colon [6], [7], [8], [9], we investigated numbers of apoptotic cells and immune cells within the colon mucosa of *C. jejuni* infected infant mice *in situ* by immunohistochemistry. Naive infant mice served as controls. At day 6 p.i., irrespective of the *C. jejuni* strain used, infected infant mice displayed a two-fold increase of apoptotic cells, paralleled by a significant increase of neutrophilic granulocytes and macrophages as compared to naive controls (**Fig. 4A–C**). In addition, both *C. jejuni* strains induced multifold increases in T-lymphocytes, regulatory T-cells (Tregs) as well as B-lymphocytes in infected versus naïve infant mice (**Fig. 4D–F**). The immune cell responses were accompanied by increased expression of pro-inflammatory cytokines and mediators in the colon. The levels of nitric oxide, TNF-α, IL-6, and MCP-1 were significantly higher in colon biopsies derived from *C. jejuni* ATCC 43431 and B2 infected mice at day 6 p.i. as compared to naive controls (**Fig. 5A–D**). For IFN-γ a significant increase was measured in *C. jejuni* B2 infected mice while *C. jejuni* ATCC 43431 infected mice displayed a trend towards elevated concentrations (**Fig. 5E**). Of note, following infection with either *C. jejuni* strain, adult mice harboring a conventional gut microbiota displayed neither clinical signs of enterocolitis nor relevant immune cell or pro-inflammatory responses in their colon (also refer to [17]). Taken together, *C. jejuni* infection of infant mice resulted in distinct pro-inflammatory responses and apoptotic changes in the colonic mucosa.

### Treatment with Live *E. coli* Abrogates Murine Colonization Resistance Against *C. jejuni*

**Figure 6:**
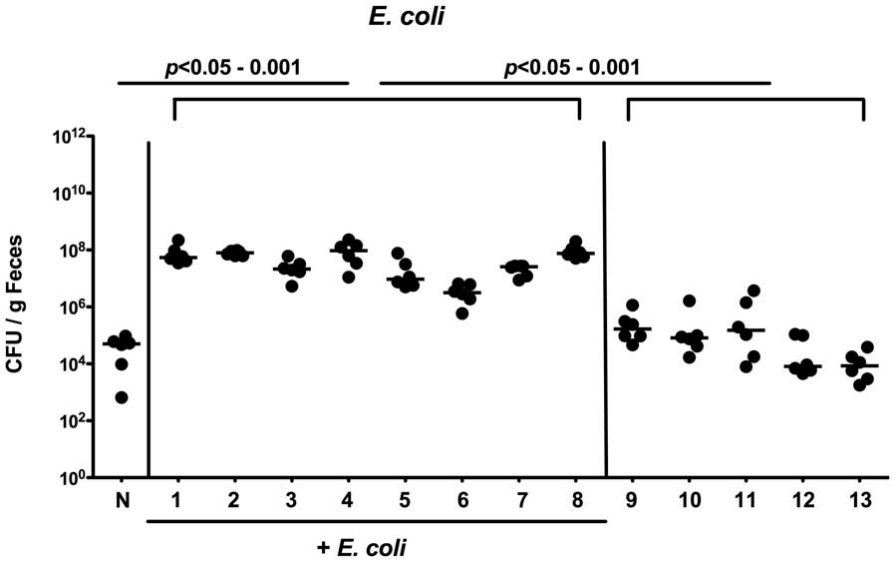
*E. coli* loads of adult mice following application of *E. coli* via drinking water. In conventionally colonized 3-months-old adult mice (n = 6) intestinal *E. coli* loads were raised by adding live *E. coli* into the drinking water (+ *E. coli*, until day 8) as described in Methods. At day 8, the *E. coli* suspension was withdrawn and replaced by regular tap water. Kinetic analyses of *E. coli* densities were performed by quantification of live bacteria in fecal samples by cultural analysis (CFU, colony forming units) until day 13 p.i. Medians (black bars) and levels of significance (*P*-values) as compared to the indicated groups (determined by Mann-Whitney-U test) are indicated. Data shown are representative for three independent experiments.

**Figure 7:**
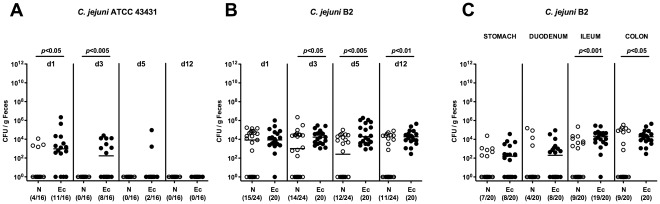
Time course of *C. jejuni* colonization in adult mice with increased *E. coli* loads. In conventionally colonized 3-months-old adult mice intestinal *E. coli* loads were raised up to 10^8^ bacteria per gram feces by adding live *E. coli* into the drinking water (+ *E. coli*) as described in Methods. Mice were orally infected with *C. jejuni* strain ATCC 43431 (**A**) or B2 (**B**). Kinetic analyses of pathogen densities were performed by quantification of live *C. jejuni* in fecal samples derived from mice with elevated (Ec; solid circles) or normal (N; open circles) *E. coli* loads by cultural analysis (CFU, colony forming units) until day 12 p.i. (**C**) *C. jejuni* B2 densities in distinct compartments of the gastrointestinal tract were determined in luminal samples taken from stomach, duodenum, ileum, and colon of mice with elevated (Ec; solid circles) or normal (N; open circles) *E. coli* loads at day 12 p.i. Numbers of animals harboring *C. jejuni* out of the total number of analyzed animals are given in parentheses. Days post infection, medians (black bars) and levels of significance (*P*-values) as compared to the respective group (determined by Mann-Whitney-U test) are indicated. Data shown are pooled from at least three independent experiments.

In order to dissect whether colonization resistance is abrogated by shifts in the microbiota composition or by conditions of inflammation we increased intestinal *E. coli* loads artificially by treating healthy adult mice with live *E. coli* in the drinking water (10^8^ CFU/mL) and investigated the susceptibility to *C. jejuni* infection. Analysis of *E. coli* in feces samples revealed that the intestinal *E. coli* loads increased by more than 3 orders of magnitude (from median 10^5^ CFU/g to 10^8^ CFU/g feces) and remained stable as long as the *E. coli* suspension was kept (**Fig. 6**). Within 24 hours after withdrawl of the *E. coli* suspension, the intestinal *E. coli* loads decreased back to basal levels (**Fig. 6**). Oral infection of *E. coli*-treated mice and non-treated controls with *C. jejuni* strains ATCC 43431 or B2 revealed that *E. coli* did significantly increase the colonization capacity of either strain. (**Fig. 7A and B**). *C. jejuni* B2 could be cultured from the entire GI tract of *E. coli* treated animals at day 12 p.i. with highest counts in distal parts such as ileum and colon (**Fig. 7C**). In non-treated control animals, however, pathogen burdens were significantly lower as compared to mice with elevated intestinal *E. coli* load (**Fig. 7**).

### Pro-inflammatory Immune Responses in *C. jejuni* Infected Adult Mice with Elevated *E. coli* Loads

**Figure 8:**
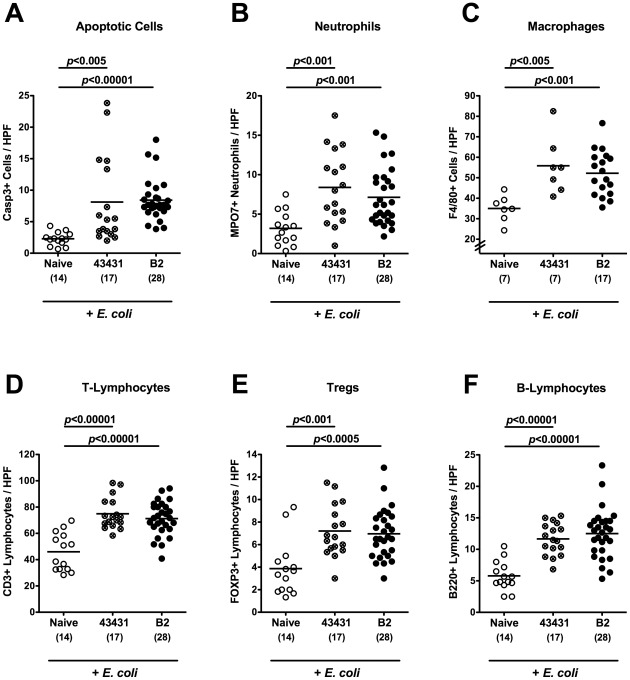
Immune responses following *C. jejuni* infection of adult mice with increased *E. coli* loads. In conventionally colonized 3-months-old adult mice intestinal *E. coli* loads were raised up to 10^8^ bacteria per gram feces by adding live *E. coli* into the drinking water (+ *E. coli*) as described in Methods. Then, mice were orally infected with *C. jejuni* strain ATCC 43431 (crossed circles) or B2 (solid circles). The average numbers of apoptotic cells (positive for caspase-3, panel **A**), neutrophilic granulocytes (Neutrophils, positive for MPO-7, panel **B**), macrophages (positive for F4/80, panel **C**), T-lymphocytes (positive for CD3, panel **D**), regulatory T-cells (Tregs, positive for FOXP3, panel **E**) and B-lymphocytes (positive for B220, panel **F**) from at least six high power fields (HPF, 400×magnification) per animal were determined microscopically in immunohistochemically stained colon sections at day 12 p.i. Uninfected mice (Naïve, open circles) served as negative controls. Numbers of analyzed animals are given in parentheses. Means (black bars) and levels of significance (*P*-values) as compared to uninfected (Naïve) control mice (determined by the Student’s *t*-test) are indicated. Data shown are pooled from at least three independent experiments.

To further elucidate whether *C. jejuni* infection of adult mice with high intestinal *E. coli* loads was accompanied by pro-inflammatory immune responses, we analyzed apoptosis and inflammatory cells in the colon mucosa by *in situ* immunohistochemistry. Irrespective of the *C. jejuni* strain analyzed, infected mice displayed significantly higher numbers of apoptotic cells, neutrophils, macrophages, T- and B-lymphocytes as well as regulatory T-cells in the colon *in situ* at day 12 p.i. as compared to non-infected *E. coli*-treated control animals (**Fig. 8**).

**Figure 9:**
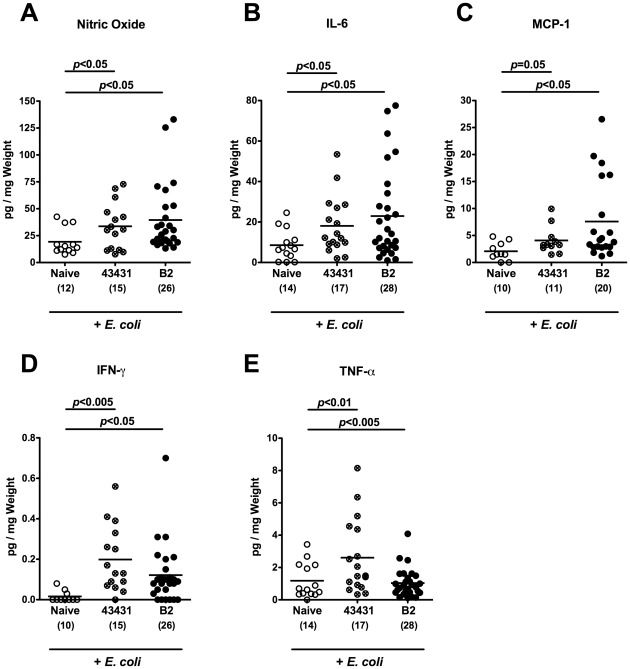
Colonic pro-inflammatory cytokine responses of *C. jejuni* infected adult mice with increased *E. coli* loads. In conventionally colonized 3-months-old adult mice intestinal *E. coli* loads were raised up to 10^8^ bacteria per gram feces by adding live *E. coli* into the drinking water (+ *E. coli*) as described in Methods. Then, mice were orally infected with *C. jejuni* strain ATCC 43431 (crossed circles) or B2 (solid circles). Concentrations (pg per mg colon) of (**A**) nitric oxide, (**B**) IL-6, (**C**) MCP-1, (**D**) IFN-γ, and (**E**) TNF-α were determined in supernatants of *ex vivo* colon cultures at day 12 p.i. by Griess reaction or CBA-ELISA, respectively. Uninfected mice (Naïve, open circles) served as negative controls. Numbers of analyzed animals are given in parentheses. Means (black bars) and levels of significance (*P*-values) as compared to the uninfected (Naïve) group determined by Student’s *t*-test are indicated. Data shown were pooled from at least three independent experiments.

Secretion of nitric oxide, IL-6, MCP-1, IFN-γ and TNF-α in *ex vivo* colon biopsies were significantly increased at day 12 following infection with *C. jejuni* ATCC 43431 or B2 versus naïve animals with supra-physiological intestinal *E. coli* loads (**Fig. 9**). Thus, *C. jejuni* infection of adult mice treated with supra-physiological *E. coli* loads is accompanied by pro-inflammatory immunopathology of the colon. Of note, elevation of intestinal *E. coli* loads alone did not affect pro-inflammatory immune responses (data not shown). This was even true in our recent study when gnotobiotic mice generated by antibiotic treatment were replenished with a complex human microbiota [17].

Taken together, physiological (infancy) and pathological conditions (e.g. acute and chronic intestinal inflammation in mice and men) associated with shifts of the intestinal microbiota composition such as elevated commensal *E. coli* loads facilitate *C. jejuni* infection inducing or perpetuating immunopathology. Most strikingly, the physiological CR against *C. jejuni* can be abrogated by elevating a single bacterial commensal gram-negative species such as *E. coli* within the complex commensal intestinal microbiota towards supra-physiological levels. These results point towards a pivotal role of *E. coli* in facilitating *C. jejuni* infection.

## Discussion

Investigations of the molecular mechanisms underlying *C. jejuni* induced immunpathology *in vivo* are hampered by colonization resistance of mice due to their host-specific intestinal microbiota composition. In the present study we investigated the susceptibility of an initially resistant host to *C. jejuni* during acute and chronic intestinal inflammation. In our acute *T. gondii-*induced ileitis model colonization resistance against *C. jejuni* was completely abolished and the pathogen colonized the entire GI tract with high counts. Moreover, *C. jejuni* and commensal *E. coli* translocated into MLNs, spleen, and liver. Strikingly, *C. jejuni* could be cultured from cardiac blood samples in >90% of infected animals displaying severe ileitis indicating bacteremia and subsequent sepsis. The impact of intestinal inflammation in overcoming colonization resistance was further supported by the finding that IL-10*^−^* ^/*−*^ mice suffering from chronic colitis were susceptible to *C. jejuni* infection. This is well in line with previous findings of Mansfield *et al.* showing that *C. jejuni* induced significant clinical signs of enterocolitis following infection of IL-10*^−^* ^/*−*^ mice [41]. However, in the IL-10*^−^* ^/*−*^ mice analyzed here the clinical signs of enterocolitis following *C. jejuni* infection remained rather subtle. The reason for this discrepancy in colitis severity is most likely attributed to differences in the genomic contents of the *C. jejuni* strains used.

The intestinal inflammation models exerting a compromised colonization resistance against *C. jejuni* had all in common significantly higher *E. coli* loads at inflamed tissue site, for instance, when compared to healthy controls with an intact colonization resistance, irrespective whether the inflammatory condition was acute or chronic, located in the small or large intestine. These findings are well in line with results from previous studies pointing towards a weakness of the physiological barrier during intestinal inflammation [19], [20], [21], [42]. Bacteremia and sepsis display hazardous sequelae of *C. jejuni* infection in hosts with a compromized epithelial barrier function (e.g. Crohn’s disease patients with acute episodes). A high prevalence of *C. concisus* was recently detected in paediatric CD as well as adult UC patients [43], [44]. Although distinct from IBD, irritable bowel syndrome was described to occur as a potential post-infectious event following *C. jejuni* infection [45]. Taken together, these findings once more point towards a correlation between inflammation, intestinal microbiota and susceptibility to *C. jejuni infection.*

Given that colonization of the intestinal tract starts right after birth and complexity increases thereafter, the question arises whether infant mice already display a fully established colonization resistance. Neonatal mice had been used in *Campylobacter* research already three decades ago, investigating transmissibility from infant to dam, virulence factors, vaccines and the efficacy of bismuth subsalicylate in preventing the growth and colonization of the intestinal tract [35], [36], [37], [39], [46]. Remarkably, infection of 3-weeks-old mice right after weaning in our study revealed that *C. jejuni* B2, and to a less distinct extent strain ATCC 43431, could stably colonize the GI tract of mice. Moreover, infant mice displayed approximately 2–3 orders of magnitude higher *E. coli* counts in their feces as compared to 3-months-old adult animals. Thus, there is evidence that an altered microbiota towards higher enterobacteria numbers (such as *E. coli*) might facilitate *C. jejuni* infection. When assessing *C. jejuni* induced clinical pathology due to blood-positive stool samples and mild diarrhea in the first week following infection, the clinical outcomes were similar in infant mice infected with *C. jejuni* strains B2 or ATCC 43431. Both strains originate from humans with severe enteritis, are invasive *in vitro* and induced distinct pro-inflammatory immune responses within the colon mucosa of *C. jejuni* infected infant mice. The elevated intestinal *E. coli* loads in mice suffering from intestinal inflammation and in infant mice provide evidence that *E. coli* is essentially involved in abrogation of CR. This is further underlined by a substantially increased colonization capacity of *C. jejuni* in adult mice in which the commensal intestinal microbiota was artificially modified towards supra-physiological intestinal *E. coli* loads. This finding demonstrates again that CR against *C. jejuni* is mediated by the microbiota and that shifts in the flora represent a risk for *C. jejuni* infection.

We conclude that the murine models of *C. jejuni* infection presented here shed further lights into the mechanisms underlying CR against *C. jejuni* offering promising tools to further unravel the interplay between intestinal pathogens, gut microbiota and the innate and adaptive immune system.

## Materials and Methods

### Ethics Statement

Animal experiments were conducted according to the European Guidelines for animal welfare (2010/63/EU) with approval of the commission for animal experiments headed by the “Landesamt für Gesundheit und Soziales” (LaGeSo, Berlin, Germany; Registration numbers: G0173/07 and G135/10). Animal welfare was monitored twice daily by assessment of clinical conditions.

### Mice

Mice were bred and maintained in the facilities of the “Forschungsinstitut für Experimentelle Medizin” (FEM, Charité - Universitätsmedizin, Berlin, Germany), under specific pathogen-free (SPF) conditions. C57BL/6 mice were used in the experiments. Age matched female mice 3 weeks (right after weaning; infant mice) and 3 months of age (adult mice) as well as 6 months old IL-10*^−^* ^/*−*^ mice (C57BL/6 background) with overt chronic colitis were used.

### Clinical Score

To assess clinical/macroscopic signs of *C. jejuni* induced infection on a daily basis, a standardized cumulative clinical score (maximum 12 points; modified according to [47] addressing the occurrence of blood in feces (0 points: no blood; 2 points: microscopic detection of blood by the Guajac method using Haemoccult, Beckman Coulter/PCD, Krefeld, Germany; 4 points: macroscopic blood visible), diarrhea (0: formed feces; 2: pasty feces; 4: liquid feces), and the clinical aspect (0: normal; 2: ruffled fur, less locomotion; 4: isolation, severely compromized locomotion, pre-final aspect) was used.

### Induction of Acute Ileitis

For induction of ileitis, C57BL/6 mice were infected orally with 100 *Toxoplasma (T.) gondii* cysts (ME49 strain) from homogenized brains of intraperitoneally infected NMRI mice in a volume of 0.3 ml phosphate-buffered saline (PBS, pH 7.4) by gavage, as described previously [22], [23], [48], [49].

### *C. jejuni* Infection of Mice

Mice were infected with approximately 10^9^ viable CFU of *C. jejuni* strains ATCC 43431 or B2 by gavage in a total volume of 0.3 mL PBS on two consecutive days as described earlier in detail [17]. *C. jejuni* strain B2 was kindly provided by Prof. Dr. Uwe Groß, University of Göttingen, Germany.

### Elevation of Intestinal *E. coli* Loads

In order to raise intestinal *E. coli* loads in 3-months-old adult mice harboring a conventional gut microbiota to supra-physiologic levels, live *E. coli* were isolated from naïve, healthy 3 months old C57BL/6 mice by culture on MacConkey media. This strain was shown to be a common commensal in healthy mice. PCR-based detection revealed that this strain does not contain known virulence factors of pathogenic *E. coli* such as stx 1 and 2, catA, hlyA, cspA, katP, and astA. This commensal *E. coli* strain was subcultivated on blood agar and added to the drinking water at a final concentration of 10^8^ CFU per mL suspension starting at day 0 in naïve animals. The *E. coli* suspension was renewed daily. For kinetic experiments, the *E. coli* suspension was withdrawn after 8 days and replaced by regular tap water thereafter. For *C. jejuni* infection studies, the application of *E. coli* in drinking water was started 8 days prior the first oral *C. jejuni* infection and continued until the end of the experiment (12 days following the latest *C. jejuni* gavage).

### Sampling Procedures, Determination of Colon Length and Histopathology

Mice were sacrificed by isofluran treatment (Abbott, Germany). From each mouse samples derived from the GI tract (stomach, duodenum, ileum, colon) were collected in parallel for histological, microbiological, immunobiological and molecular analyses. Cardiac blood and tissue samples from mesenteric lymphnodes, spleen, liver and the GI tract were asserved under sterile conditions. Histopathological changes were determined in colon samples immediately fixed in 5% formalin and embedded in paraffin. Sections (5 µm) were stained with respective antibodies for immunohistochemistry.

### Immunohistochemistry

*In situ* immunohistochemical analysis of colonic paraffin sections were performed as described previously [17], [24], [50]. Primary antibodies against CD3 (#N1580, Dako, Denmark, dilution 1∶10), FOXP-3 (FJK-16s, eBioscience, 1∶100), B220 (eBioscience, San Diego, CA, USA, 1∶200), myeloperoxidase-7 (MPO-7, # A0398, Dako, 1∶400), F4/80 (#14–4801, clone BM8, eBioscience, 1∶50), and cleaved caspase-3 (Asp175, Cell Signaling, USA, 1∶200) were used. For each animal, the average number of positive stained cells within at least six independent high power fields (HPF, 400×magnification) were determined microscopically and subjected to statistical analysis as indicated.

### Cytokine Detection in Colon Culture Supernatants

Colon biopsies were cut longitudinally, washed in PBS and strips of 1 cm^2^ placed in 24-flat-bottom well culture plates (Nunc, Wiesbaden, Germany) containing 500 µl serum-free RPMI 1640 medium supplemented with penicillin (100 U/ml) and streptomycin (100 ug/ml; PAA Laboratories). After 18 h at 37°C, culture supernatants were tested for TNF-α, IL-6, MCP-1 and IFN-γ by the Mouse Inflammation Cytometric Bead Assay (CBA; BD Biosciences) on a BD FACSCanto II flow cytometer (BD Biosciences). Nitric oxide (NO) was determined by Griess-reaction as described earlier [22].

### Analysis of the Intestinal Microflora

Cultural analyses, biochemical identification and molecular detection of luminal bacterial communities from stomach, duodenum, ileum, and colon as well as feces were performed as previously described [17], [22], [23], [24].

### Assessment of Bacterial Translocation Frequencies

For bacterial translocation experiments, mesenteric lymphnodes (MLNs) draining the small intestine, spleen, liver (1 cm^2^) and cardiac blood (1 mL) were transferred into thioglycollate enrichment broths under sterile conditions and cultivated for 7 days. Turbid broths were streaked onto sheep blood, MacConkey as well as Karmali agar in order to detect translocated live *E. coli* and *C. jejuni* followed by biochemical species identification (API 20E and API Campy, respectively, Biomérieux). Given that bacterial growth in enrichment broths cannot be quantitated, the relative rates of translocating *C. jejuni* or *E. coli* into the respective extra-intestinal compartment are expressed in % (number of broths culture-positive for either *E. coli* or *C. jejuni* multiplied with 100 and divided by the total number of broths inoculated with the respective organ or blood).

### Statistical Analysis

Mean values, medians, standard deviations, and levels of significance were determined using appropriate tests as indicated (two-tailed Student’s *t*-Test, Mann-Whitney-U Test). Two-sided probability (*P*) values ≤0.05 were considered significant. All experiments were repeated at least twice.
